# Motility characteristics in the transition zone in Gastroesophageal Reflux Disease (GORD) patients

**DOI:** 10.1186/s12876-016-0525-1

**Published:** 2016-08-30

**Authors:** Yu-wen Li, Chen-xi Xie, Kai-Ming Wu, Min-hu Chen, Ying-lian Xiao

**Affiliations:** 1 Department of Gastroenterology, First affiliated Hospital, Sun Yat-sen University, Guangzhou, GuangDong Province 510080 People’s Republic of China; 2 Department of Gastroenterology & Hepatology, Princess Alexandra Hospital, The University of Queensland, Brisbane, QLD 4102 Australia; 3 Gastrointestinal Surgery Center, First affiliated Hospital, Sun Yat-sen University, Guangzhou, GuangDong Province 510080 People’s Republic of China

**Keywords:** Gastroesophageal reflux disease, High-resolution Impedance manometry, Transition zone, Bolus transit

## Abstract

**Background:**

Defects in distal oesophageal peristalsis was thought to be an indication of incomplete bolus transit (BT). However, the role of transition zone (TZ) defects in the BT in gastroesophageal reflux disease (GORD) patients needs clarification. The aim of this study was to assess the TZ defects in GORD patients and to explore the relationship between TZ defects and BT.

**Methods:**

One hundred and two patients with reflux symptoms and 20 healthy adults were included in the study. All subjects underwent upper gastrointestinal endoscopy, high resolution impedance manometry (HRiM) and 24-h ambulatory multichannel impedance-pH (MII-pH) monitoring. Patients were subgrouped into reflux oesophagitis (RE), non-erosive reflux disease (NERD), hypersensitive oesophagus (HO) and functional heartburn (FH) classified following MII-pH monitoring. Oesophageal pressure topography was analysed to define TZ defects by spatial or temporal TZ measurements exceeding 2 cm or 1 s, weak and fragmented swallows were excluded, and the association between TZ and BT was investigated.

**Results:**

Following liquid swallows, there were no significant differences in TZ delay time and TZ length between groups (RE: 1.75 s (1.32–2.17) and 2.50 cm (2.40–3.20); NERD: 1.60 s (1.10–2.00) and 2.20 cm (2.10–2.65); HO: 1.60 s (1.30–1.80) and 2.70 cm (2.30–3.00); FH: 1.55 s (1.20–2.17) and 3.10 cm (2.25–5.00); Healthy volunteers: 1.50 s (1.20–1.90) and 2.30 cm (2.10–3.00). However, individuals with TZ defects had lower complete BT rates compared with those without TZ defects (*p* < 0.001). There were also significantly more incomplete BT in patients with RE, HO and FH than in healthy controls (*p* < 0.05).

**Conclusions:**

In GORD patients, TZ defects correlated with proximal bolus retention in the corresponding area independent of distal weak peristalsis.

**Electronic supplementary material:**

The online version of this article (doi:10.1186/s12876-016-0525-1) contains supplementary material, which is available to authorized users.

## Background

Gastroesophageal reflux disease (GORD) is common and develops when reflux of gastric contents causes uncomfortable symptoms and/or complications [1]. The prevalence of GORD is increasing both in western countries and Asia due to the increasing obesity epidemic [2, 3]. It has been reported that severe oesophagitis (Los Angeles grading C, D) healed without any improvement of motor function in the oesophageal body following acid suppression therapy, so oesophageal dysmotility may be regarded as a primary factor in the pathogenesis of reflux oesophagitis (RE), particularly if RE is severe [4]. High-resolution oesophageal manometry (HRM) is considered an important clinical tool in assessing motor function of the tubular oesophagus. Using this technique, peristalsis defects including defects located in both the transition zone (TZ) and distal oesophagus may be identified. Histological studies have shown that the human oesophagus is composed of two types of muscle. The proximal one-third is composed of striated muscle, while the distal two-thirds comprises smooth muscle. Previous studies have suggested that bolus transport requires the spatiotemporal coordination between proximal and distal contractile waves [5, 6]. The area between these two contractile waves is called the transition zone (TZ). Manometric studies have documented that the TZ (also called low-pressure zone), is situated adjacent to the aortic arch and carina [7, 8].

With the aid of high-resolution impedance manometry (HRiM) analysis, the effect of contractile patterns on transit can be assessed [9]. Roman and colleagues have found that distal oesophageal defects are related to bolus retention in the distal oesophagus in GORD patients and that dysphagia symptoms were significantly correlated with bolus retention [10]. Two other studies also showed that TZ defects were associated with BT across the TZ [11, 12]. In addition, TZ defects have been shown to be related to dysphagia [13–15], globus [16] and PPI non-responsers [17]. TZ defects have even been considered as distinct oesophageal motility disorders [13]. Pharyngeal failed swallows or impaired clearance can be detected in patients with pharyngeal dysphagia [18]. Several studies have shown that ineffective oesophageal motility (IEM) and/or nontransmitted proximal contractions are frequently observed in GORD patients [19, 20]. However, whether the TZ defects are correlated with the bolus retention in GORD patients has not yet been studied.

IEM is the primary motility disorder in GORD patients. The recently published Chicago classification of oesophageal motility disorders 3.0 [21] adopted a brand-new concept of IEM using the distal contractile integral (DCI), which replaced the traditional definition of IEM which used the mean oesophageal contractile amplitude in conventional line tracing manometry. However, both the current and previous definition of IEM focuses on the distal peristalsis integrity. The clinical significance of TZ defects in GORD has not been investigated.

Thus the aim of the current study was to assess TZ defects and its relation to bolus retention in GORD patients.

## Methods

### Study design

Consecutive patients presenting to Gastroenterology Outpatients for recurrent heartburn and/or regurgitation (≥2 days/week) were included. The Gastroesophageal Reflux Disease Questionnaire (GERD Q) was used to evaluate patient symptoms. Only patients with reflux symptoms for > 3 months and at least moderate symptom intensity scores were eligible. Patients were excluded if they: 1) had peptic ulcers, erosive gastritis, or gastrointestinal tumors detected through endoscopy; 2) were unable to complete all 3 procedures; 3) were women complicated by pregnancy; 4) had severe systemic disease.

All patients underwent upper gastrointestinal endoscopy, HRiM and 24-h ambulatory multichannel impedance-pH (MII-pH) monitoring after at least one week of pharmacological washout of acid suppression therapy.

Twenty healthy volunteers (control subjects), without any gastrointestinal symptoms or history of relevant GI disease or recent gastrointestinal medication during the previous 3 months were recruited via advertisement. Following the clinical assessment, control subjects underwent all diagnostic procedures. Signed informed consent was obtained from all participants in the study prior to any procedure.

### High-Resolution Impedance Manometry (HRiM)

The HRiM system (Sandhill Scientific, Inc., Highland Ranch, CO, USA) was used to capture oesophageal pressure. Following the relevant calibration procedures, a catheter with 32 circumferential solid-state pressure sensors (4.2 mm in diameter) located at 1 cm apart and 5 dual impedance measuring sensors (5 cm apart) was inserted through a nostril after pressure and impedance calibration. After recording oesophageal baseline characteristics in each patient whilst in the recumbent position, 10 standardized liquid swallows (5 ml saline) and 10 viscous swallows (5 ml yoghourt) at 20 s intervals were undertaken and recorded.

### 24-hour ambulatory multichannel impedance-pH Monitoring

Combined multichannel intraluminal impedance and pH (MII-pH) monitoring was undertaken (Sandhill Scientific Inc., Highlands Ranch, CO, USA). The Impedance probe was positioned with the proximal pH electrode 5 cm above lower oesophageal sphincter (LOS) as localized by HRiM. The 6 impedance measuring sensors were located in 3,5, 7, 9, 15 and 17 cm above the LOS. Patients were asked to record meal times, whether they were ambulatory or supine and symptom occurrence. Pathologic oesophageal acid reflux (pH(+)) was identified if the total percentage time of oesophageal pH values below 4 exceeded 4 %.

Patients were categorized into the following groups: RE (presence of mucosal lesions identified on upper endoscopy according to the Los Angeles Classification), NERD (erosions absent but pathologic oesophageal acid reflux positive); HO (hypersensitive oesophagus) (positive symptom association probability score (SAP) to acid reflux and physiological acid exposure); FH functional heartburn (patients with no mucosal lesion on upper endoscopy, negative 24-h ph-impedance monitoring and negative SAP) [22].

### Data analysis

#### HRiM

All the oesophageal pressure topography was analyzed manually using BioView Analysis software (Sandhill Scientific Inc., Highlands Ranch, CO, USA). All failed swallows, fragmented swallows and weak swallows were excluded. The integrated relaxation pressure (IRP), distal contractile integral (DCI), and distal latency (DL) were calculated as previously described [23]. The complete BT as defined by Simren and colleagues [24] was used, i.e. bolus entry at a 50 % drop in impedance from baseline to nadir and bolus exit at the recovery of impedance to the 50 % value.

#### Identification of TZ

**Fig. 1:**
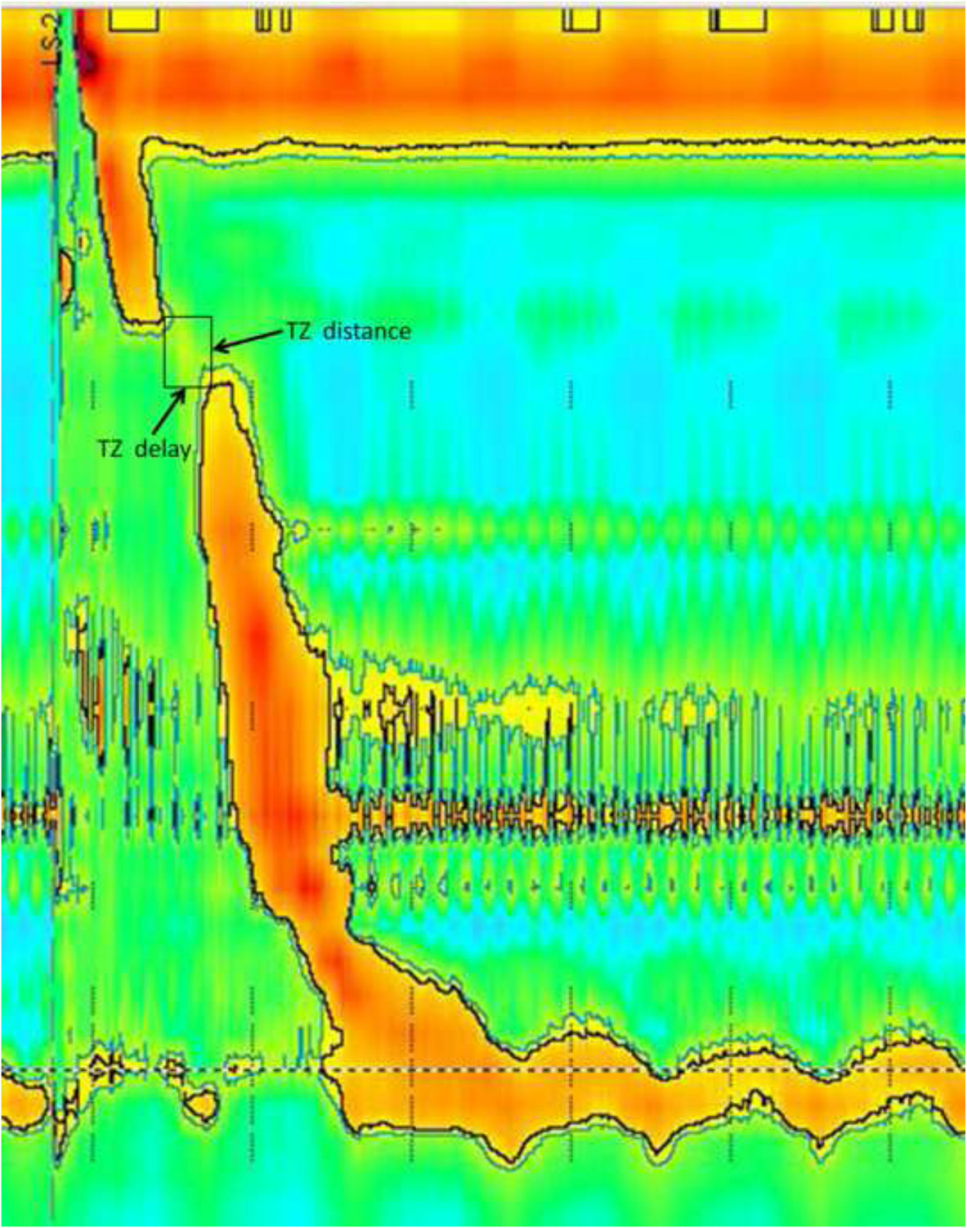
Measurement of the oesophageal transition zone (TZ). The black line depicts the 20 mmHg isocontour line

TZ was identified using the 20 mmHg isobaric contour [13]. Both the spatial and temporal distance were recorded in the TZ measurement (Fig. 1). Normal TZ ranges were defined using data obtained from the healthy volunteers with an abnormality defined as exceeding the 95^th^ percentile of the normal subjects (2 cm, 1 s) [6].

### Statistical analysis

HRiM parameters were represented as medians and interquartile ranges. The Kruskal-Wallis test was used to compare parameters. SPSS 17.0 (SPSS, Inc., Chicago, IL, USA) software was used to perform the statistical analysis. Two-tailed P value was considered statistically significant if less than 0.05.

## Results

### Demographic characteristics

A total of 102 patients were recruited into the study. 3 were excluded due to abnormal findings on upper endoscopy. Among the 20 healthy volunteers, 4 were excluded due to artefacts in either HRiM or 24-h MII-pH monitoring. Thus, 99 patients with reflux symptoms (59 males and 40 females, mean age: 46.60 ± 13.32 years, mean body mass index [BMI]: 22.68 ± 3.18) and 16 healthy adults (7 males and 9 females, mean age: 38.94 ± 13.20 year, mean body mass index [BMI]: 21.57 ± 2.41 were included in the final analysis. Among the 99 patients, there were 38 RE, 15 NERD, 9 hypersensitive oesophagus, 37 FH patients (Table 1).

**Table 1:** Subject demographics

	RE (*n* = 38)	NERD (*n* = 15)	HO (*n* = 9)	FH (*n* = 37)	HV (*n* = 16)
Gender (F:M)	4:34	9:6	4:5	12:25	9:7
Age (mean [95 % CI])	48.03 ± 15.53	50.33 ± 14.25	41.33 ± 14.49	44.89 ± 9.55	38.94 ± 13.20
BMI (mean [95 % CI])	23.35 ± 3.06	23.64 ± 2.47	23.78 ± 3.01	21.35 ± 3.25	21.57 ± 2.41
pH(+):pH(−)	23:15	—	—	—	—

*RE* reflux oesophagitis, *NERD* non-erosive reflux disease, *HO* hypertensive oesophagus, *FH* functional heartburn, *HV* healthy volunteer

As failed swallows, weak swallows and fragmented swallows were excluded, in total 154 liquid swallows and 144 viscous swallows from healthy volunteers and 723 liquid swallows and 601 viscous swallows from patients were included. When broken down into the subject groups, the following swallows were recorded: RE, 268 liquid, 206 viscous swallows; NERD, 118 liquid, 98 viscous swallows; HO, 70 liquid, 65 viscous swallows; and for FH 267 liquid, 232 viscous swallows.

### Comparison of high-resolution manometry impendence parameters

Table 2 shows a comparison of HRiM results when all the swallows (including failed, weak, fragment and intact swallows) were included in the analysis. Patients with GORD had lower IRP, and lower DCI than healthy volunteers, both in liquid swallows and viscous swallows.

**Table 2:** Comparison of High-resolution Manometry-Multichannel Impedance Parameters in subject groups for liquid and viscous swallows (including failed, weak and fragment swallows)

		RE	NERD	HO	FH	HV	*P* values
Liquid swallows	IRP (mmHg)	10.3 (14.4–19.10)	11.50 (6.70–14.60)	11.23 (8.65–14.08)	14.30 (12.13–17.90)	14.15 (11.10–20.52)	0.000
	DCI (mmHg · s · cm)	988 (510–1532)	927 (587–1651)	918 (525–1465)	1112 (644–1548)	1240 (933–1744)	0.000
	DL (s)	6.4 (5.80–7.10)	6.20 (5.60–7.13)	6.10 (5.50–6.65)	6.60 (6.00–7.30)	6.60 (6.00–7.10)	0.000
	TZ delay (s)	1.30 (1.80–2.30)	1.60 (1.10–2.00)	1.70 (1.30–2.45)	1.80 (1.30–2.50)	1.50 (1.25–2.10)	0.142
	TZ distance (cm)	2.30 (2.50–4.10)	2.20 (2.60–3.08)	2.95 (2.37–4.45)	3.20 (2.40–5.20)	2.50 (2.20–3.60)	0.140
viscous swallows	IRP (mmHg)	11.90 (8.38–17.00)	9.40 (6.90–12.80)	9.85 (7.28–15.45)	12.80 (9.60–15.00)	12.45 (8.70–18.23)	0.000
	DCI (mmHg · s · cm)	720 (184–1413)	766 (426–1525)	651 (210–1484)	927 (333–1565)	1104 (788–1542)	0.000
	DL (s)	7.00 (6.30–7.70)	7.00 (5.80–8.35)	6.70 (5.90–7.60)	7.30 (6.60–8.40)	7.40 (6.80–8.10)	0.000
	TZ delay (s)	1.45 (2.00–2.70)	2.00 (1.40–2.68)	1.90 (1.40–2.60)	2.00 (1.40–2.63)	2.00 (1.50–2.50)	0.922
	TZ distance (cm)	2.80 (2.50–5.75)	4.40 (2.63–6.13)	3.00 (2.25–4.58)	3.20 (2.40–5.85)	3.15 (2.53–4.68)	0.580

*RE* reflux oesophagitis, *NERD* non-erosive reflux disease, *HO* hypertensive oesophagus, *FH* functional heartburn, *HV* healthy volunteer, *IRP* integrated relaxation pressure, *DCI* distal contractile integral, *DL* distal latency, *TZ* transitional zone

Data are presented as median (interquartile range)

When failed, weak and fragment swallows were excluded (Table 3), for liquid swallows, the patients with GORD had lower IRP, and lower DCI than healthy volunteers. In terms of the viscous swallows, the patients with GORD had lower IRP, lower DCI and longer DL than healthy volunteers.

**Table 3:** Comparison of High-resolution Manometry-Multichannel Impedance Parameters in subject groups for liquid and viscous swallows

		RE	NERD	HO	FH	HV	*P* values
Liquid swallows	IRP (mmHg)	14.65 (10.00–19.87)	12.10 (7.30–15.20)	10.95 (8.28–14.48)	14.90 (12.50–18.70)	14.75 (11.97–21.65)	0.000
	DCI (mmHg · s · cm)	1210 (791–1705)	1078 (719–1745)	1021 (823–1789)	1261 (935–1703)	1283 (989–1837)	0.042
	DL (s)	6.40 (5.72–7.10)	6.25 (5.60–7.22)	6.10 (5.70–6.60)	6.60 (6.00–7.20)	6.50 (6.00–6.92)	0.001
	TZ delay (s)	1.75 (1.32–2.17)	1.60 (1.10–2.00)	1.60 (1.30–1.80)	1.55 (1.20–2.17)	1.50 (1.20–1.90)	0.437
	TZ distance (cm)	2.50 (2.40–3.20)	2.20 (2.10–2.65)	2.70 (2.30–3.00)	3.10 (2.25–5.00)	2.30 (2.10–3.00)	0.082
viscous swallows	IRP (mmHg)	12.00 (8.30–16.85)	10.15 (7.17–12.82)	10.90 (7.35–15.65)	13.10 (9.83–15.88)	13.10 (9.50–18.73)	0.000
	DCI (mmHg · s · cm)	1186 (802–1844)	1078 (699–1736)	1163 (651–1758)	1257 (834–1926)	1202 (894–1646)	0.242
	DL (s)	7.00 (6.38–7.93)	6.90 (5.80–8.48)	6.70 (5.75–7.65)	7.25 (6.60–8.30)	7.40 (6.83–8.10)	0.000
	TZ delay (s)	1.40 (1.80–2.40)	1.50 (1.30–2.00)	1.65 (1.35–2.00)	1.80 (1.30–2.38)	1.40 (1.80–2.10)	0.634
	TZ distance (cm)	2.40 (2.60–2.70)	2.20 (2.10–2.30)	2.65 (2.10–3.03)	2.60 (2.20–3.10)	2.80 (2.35–4.25)	0.204

*RE* reflux oesophagitis, *NERD* non-erosive reflux disease, *HO* hypertensive oesophagus, *FH* functional heartburn, *HV* healthy volunteer, *IRP* integrated relaxation pressure, *DCI* distal contractile integral, *DL* distal latency, *TZ* transitional zone

Data are presented as median (interquartile range)

When weak, distal large break and fragmented swallows, were excluded, there were no significant differences in TZ delay and TZ length for both liquid swallows and viscous swallows amongst RE, NERD patients, FH patients and healthy adults (Table 3).

**Fig. 2:**
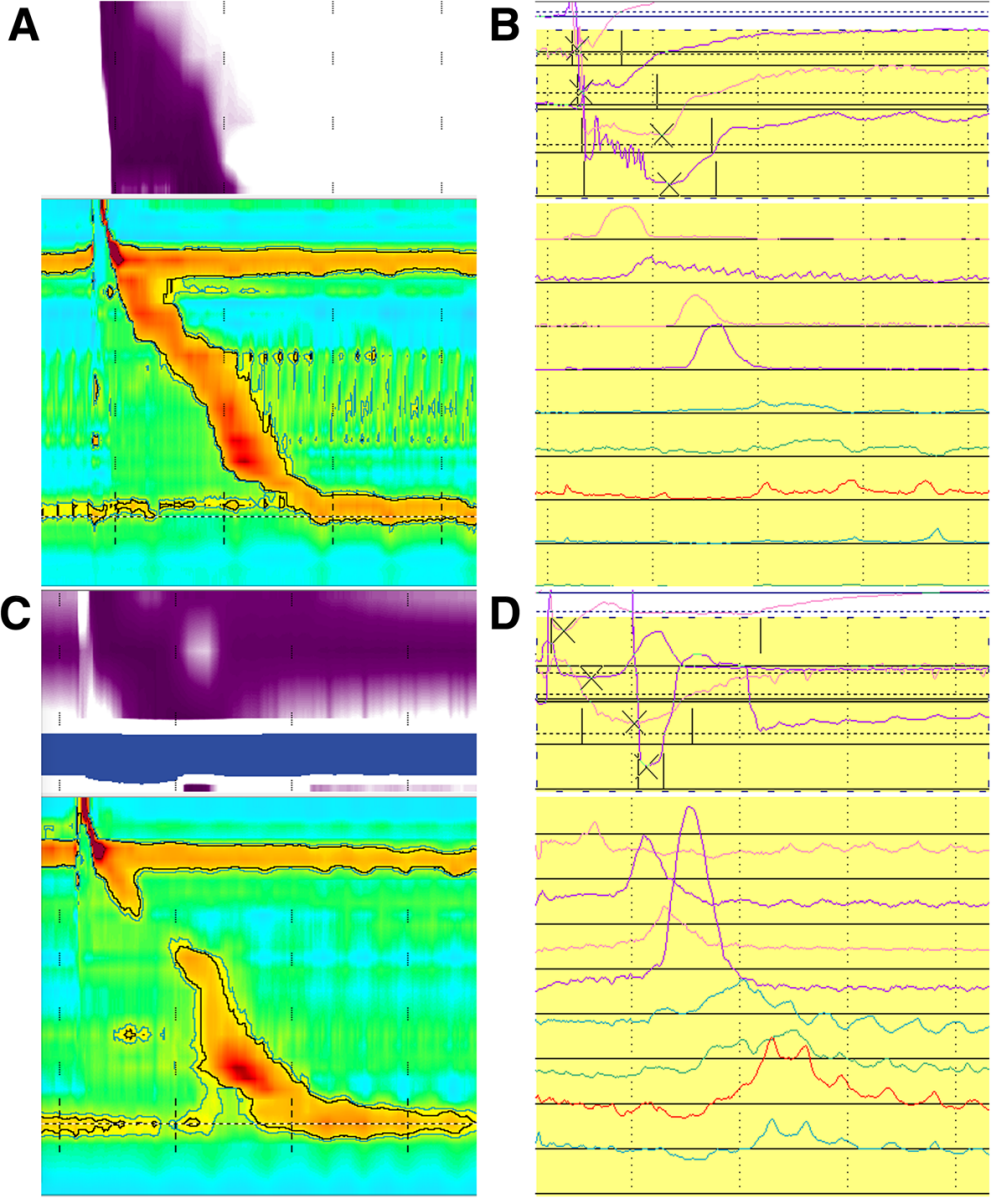
Pressure topography and impedance of manometry plots of patients with and without transition zone (TZ) defects. **a** Oesophageal pressure topography plot of a patient without a transition zone (TZ) defect: the bolus was transited completely. **b** The impedance manometry plot of a patient without a transition zone (TZ) defect: complete bolus transited. **c** Oesophageal pressure topography plot of a patient with a transition zone (TZ) defect, incomplete bolus was not transited. **d** The impedance manometry plot of a patient with a transition zone (TZ) defect, the bolus was not transited completely

**Fig. 3:**
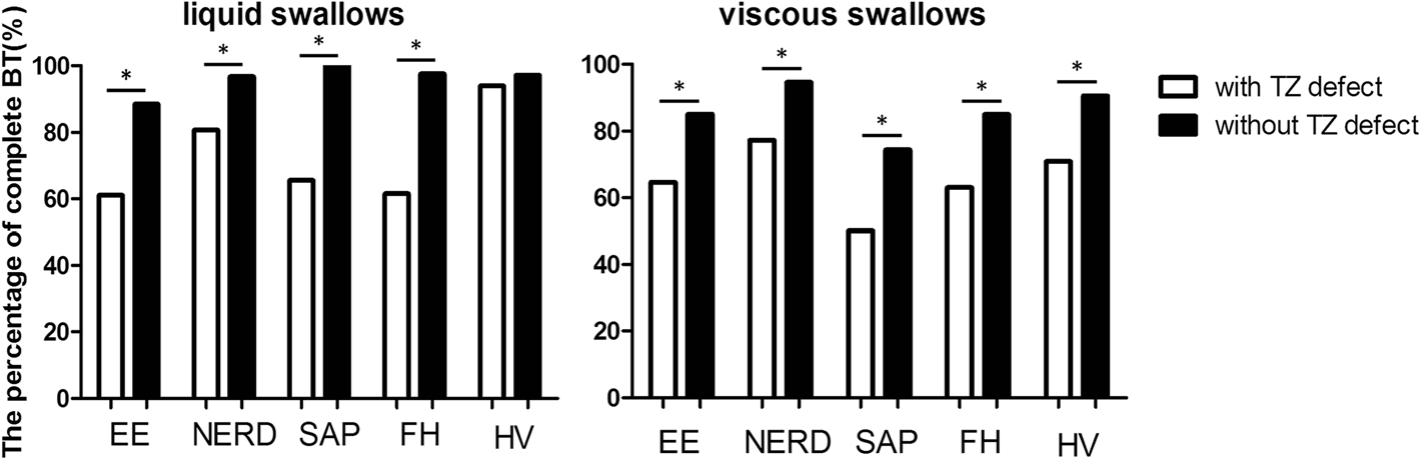
The comparasion of complete bolus transit (BT) in patients with and without transition zone (TZ) defects. The percentage of complete bolus transit (BT) of liquid swallows in patients with and without transition zone (TZ) defects. *p < 0.05; The percentage of complete bolus transit (BT) of viscous swallows in patients with and without transition zone (TZ) defects. *p < 0.05. RE, esophagitis; NERD, non-erosive reflux disease; HO, hypertensive oesophagus; FH, functional heartburn; HV, healthy volunteer

The relationship between the TZ defects and the complete BT of the second impedance channel was compared. It was found that individuals with TZ defects had lower complete BT compared with those without TZ defects (*p* < 0.001) in both liquid swallows and in viscous swallows (Figs. 2 and 3).

After weak swallows, distal large break swallows and fragmented swallows, both in liquid and in viscous swallows were excluded, the individuals with TZ defects had lower complete BT compared with those without TZ defects (*p* < 0.001). This indicated that TZ defects were related to proximal bolus retention. For liquid swallows, amongst patients with TZ defects, the complete BT rates in RE, NERD, hypersensitive oesophagus, FH patients and healthy volunteers were 61.02, 80.76, 65.52, 61.54, 94 % respectively. For viscous swallows, the complete BT rates were 64.62, 77.27, 50, 63.08, 70.84 % respectively (Fig. 3) (See in Additional file 1).

For patients with TZ defects, there were no significant correlations between BT time and TZ delay or TZ distance, and there was also no significant correlation between AET and TZ delay or TZ distance.

## Discussion

The inability of oesophageal body clearance is important in the pathogenesis of GORD, and the IEM is the primary motility disorder in GORD. The aim of the current study was to investigate the TZ defects in GORD patients. Our results showed that the TZ defects were not a discriminate factor between GORD patients and healthy volunteers, however, the TZ defects correlated to proximal bolus retention as indicated by incomplete BT in the corresponding area, independent of distal peristalsis breaks.

Ineffective swallows have been defined as the mean oesophageal contraction amplitude of less than 30 mmHg in the conventional line tracing manometry [25]. In HRM, the ineffective swallow has been re-defined in the Chicago classification v3.0 as the DCI of the swallow less than 450 mmHg.s.cm [21, 26]. Although this new definition has abandoned the concept of a peristalsis defect, the break in oesophageal body peristalsis wave has been proved to be related to the incomplete BT, which indicates the inability to clear the bolus in the oesophageal body [27]. Although some studies have proposed that intraluminal impedance can detect failure of pharyngeal bolus clearance during swallowing in patients with pharyngeal dysphagia [18], some researchers have shown that in GORD patients, non-transmitted proximal contractions are frequently observed [19, 20]. In addition, patients with large-breaks, either at the distal or proximal oesophagus, had longer bolus clearing times in supine position than patients with normal numbers of peristaltic breaks [19]. Few studies have to date explored the role of TZ defects in GORD pathogenesis.

Previous studies have shown that there were more distal peristalsis defects in GORD patients (both RE and NERD) than in healthy volunteers [4], thus the IEM were more common in GORD patients, and IEM was also the primary motility disorder found. However, in the current study, we found that the prevalence of TZ defects were similar amongst patients with RE, NERD, hypersensitive oesophagus and healthy volunteers. Some might think this would be evidence for the hypothesis that TZ defects have no role in motility abnormalities of GORD. This was shown not to be the case if the physiology of peristalsis in the BT was considered. The bolus was pushed into the stomach through the contraction of both striated and smooth muscle in the oesophagus. The motility disorders in the oesophageal body as classified by the Chicago classification focuses on the distal oesophagus, however, there is no classification relating to the function of striated muscle. What’s more, little is known of the coordination between striated muscle and smooth muscle at the location of the TZ. Thus the defect in the TZ might be a novel motility abnormality which needs to be defined in future versions of the Chicago classification.

The definition of a weak swallow has been derived from the analysis of the distal part of a single swallow, which was not related to TZ. The break of in the distal trough in the swallow in the EPT has been shown to be well correlated with the BT in the 20 mmHg isobaric contour [10]. Roman et al. have revealed that large breaks (>5 cm) were associated with incomplete BT in 100 % of instances, while small breaks (2–5 cm) were associated in only 16 % [10]. In the current study, weak swallows were omitted to avoid the confounding effect of breaks in the distal trough. Nevertheless the TZ defect was well correlated to the incomplete BT in the intact swallow, which indicated that TZ defects were independent factors for the BT in spite of the intact distal trough in a single swallow.

The TZ defines the area where the skeletal muscle and smooth muscle meet. Prior studies have demonstrated that a time delay in the TZ might be a meaningful variable in symptomatic GORD in 30 mmHg isobaric contour pressure [14]. Another study has reported that TZ defects greater than 2 cm in distance and 1 s in duration were forcefully related with unexplained dysphagia in 20 mmHg isobaric line, with an occurrence rate of 57 % in 25 dysphagia patients [13]. In our study, following liquid swallows, a significant difference between the complete BT rate with and without TZ defects was found in RE, NERD patients and also in healthy volunteers. Patients with TZ defect had lower complete BT rates than those without a TZ defect, which suggested that TZ defects had an important impact on proximal bolus retention. This was confirmed by the correlation between a TZ defect and incomplete BT. Bolus retention is related to the oesophageal symptoms, and distal bolus retention has been confirmed to be related to GORD, however we believe that the current study is the first to show proximal retention in GORD patients. The bolus retention could be related to the inability of bolus clearance in the oesophagus. In the clinical setting, this could be the reason for dysphagia. When applied to GORD, this could be an indicator of oesophageal body dysfunction. In the current study, we focused on assessing TZ defects and their relation to bolus retention. Whether this would play a role in the GERD pathogenesis or symptom genesis is unknown.

There are some limitations in the current study. Firstly, an additional group of patients with dysphagia could have been included as a comparison to evaluate the relationship of TZ defects with dysphagia symptoms, because non-obstructive dysphagia is the most common indication for HRM, and the clinical significance between TZ defects and dysphagia should be evaluated. Secondly, a more detailed record of reflux symptoms including frequency and severity would be helpful in defining the relationship between the size of TZ defects and GORD symptoms. Finally, the sample size of the healthy volunteers was relatively small. An interesting finding in our study was that our RE patients had relative high IRP compared to previously reported studies, however it should be noted that the current study used a Sandhill manometry system. where a higher IRP cut-off value has been previously reported [23].

## Conclusion

The current study has explored the role of TZ defects in complete BT, which is an important area recently attracting increasing attention in patient management of GORD. Through the analysis of the TZ defects and BT, we found that TZ defects were not a discriminatory factor between GORD and healthy volunteers, however importantly, TZ defects were correlated to the proximal bolus retention. The anatomy and physiology of the TZ has not been fully understood, and further study is needed to clarify its significance in GORD patients.

## Additional file

Additional file 1: The percentage of BT in patients with and without TZ defect. Description of data: The dataset displayed how many patients had complete or incomplete BT in patients with or without TZ defect in different groups. (XLSX 12 kb)

## Abbreviations

BT: Bolus transit
DCI: Distal contractile integral
DL: Distal latency
FH: Functional heartburn
GORD: Gastroesophageal reflux disease
HRiM: High-Resolution Impedance Manometry
IEM: Ineffective oesophageal motility
IRP: Integrated relaxation pressure
MII-pH: 24-h ambulatory multichannel impedance pH
NERD: Non-erosive Reflux Disease
RE: Reflux oesophagitis
SAP: Symptom association probability
TZ: Transition zone
